# Is fear of childbirth related to the woman’s preferred location for giving birth? A Dutch low‐risk cohort study

**DOI:** 10.1111/birt.12456

**Published:** 2019-09-24

**Authors:** Anne‐Marie Sluijs, Marc P.H.D. Cleiren, Jan M.M. van Lith, Barbro Wijma, Klaas Wijma

**Affiliations:** ^1^ Department of Obstetrics Leiden University Medical Center Leiden The Netherlands; ^2^ Faculty of Social Sciences Honours College Leiden University Leiden The Netherlands; ^3^ Unit of Gender and Medicine Department of Clinical and Experimental Medicine Linköping University Linköping Sweden; ^4^ Unit of Medical Psychology Department of Clinical and Experimental Medicine Linköping University Linköping Sweden

**Keywords:** fear of childbirth, home birth, place of giving birth

## Abstract

**Background:**

In The Netherlands, women with low‐risk pregnancy are routinely given the option of home birth, providing a unique opportunity to study the relationship between fear of childbirth (FOC) and preference for childbirth location, and whether women experience higher FOC when the actual location differs from their preference.

**Methods:**

In this prospective cohort study, 331 nulliparous and parous women completed a questionnaire at gestational week 30 (T1) and two months postpartum (T2). FOC was assessed using versions A (T1) and B (T2) of the Wijma Delivery Expectancy/Experience Questionnaire (W‐DEQ).

**Results:**

At T1, women who preferred home birth had significantly lower FOC compared with women who preferred a hospital birth (mean ± SD W‐DEQ scores: 55 ± 19.8 and 64 ± 18.3, respectively, *P* < .01). About 28% of women who responded at T2 gave birth at home. Congruence between the preferred and actual childbirth location was not predictive of FOC assessed at T2 when adjusted for obstetric and psychological variables. In an extended analysis, we found that except for prepartum FOC, the following variables also correlated with postpartum FOC: being referred because of complications and poor neonatal condition.

**Conclusions:**

Compared to women who prefer hospital birth, women who prefer home birth have lower prepartum and postpartum FOC. Giving birth at a location other than the preferred location does not appear to affect postpartum FOC. Whether giving birth at home or in the hospital, caregivers should pay extra attention to women with high FOC because they are vulnerable to postpartum FOC, especially after a complicated birth and referral.

## 1 INTRODUCTION

In most Western countries, most pregnant women choose to give birth in a hospital, even in the case of a low‐risk pregnancy. In contrast, in The Netherlands approximately half of all pregnant women, being low‐risk, are given the option of choosing either home birth or a hospital birth under the care of a midwife.^1^ Although, of all births, the percentage of home births declined in the past decade from nearly 30% to approximately 13%,^1^ this rate is still relatively high compared with other Western countries, in which only 0.5%‐2.2% of births occur at home.^2^

In The Netherlands, women with a low‐risk pregnancy typically receive their prepartum to postpartum care from a trained, licensed midwife. However, the woman can be referred to an obstetrician if the mother and/or child's risk profile changes, for example, as a result of pregnancy‐induced hypertension, prolonged labor, or postpartum hemorrhage. In addition, women who remain low‐risk but request pain relief during labor are also referred to an obstetrician. Referral during a home birth involves handing over the responsibility from the midwife to an obstetrician and transport to the hospital.

The choice of a home birth is in most Western countries considered as unusual and “alternative.” Those women are often well educated, are older,^3^, ^4^, ^5^ want to have control and continuity of care,^6^ and are less anxious about birth.^4^ In The Netherlands, when having low risk for complications, the option of home birth has been considered as normal for a long time, especially in rural regions. Here, home birth preference is related to the confidence of family and friends in home birth,^7^, ^8^ higher education (in highly urbanized areas),^9^ and the wish to remain in familiar surroundings at home and the need for personal autonomy.^10^ Factors associated with a preference for hospital delivery are the expected safety in the hospital and the wish to minimize risks.^11^, ^12^

The experiences of family and friends and birth stories in social media can influence the woman's ideas about giving birth.^7^, ^13^, ^14^ The magnitude of that influence depends largely on the woman's trust in her own capabilities and her general response to uncertain situations, which—in the case of anxiety—can reveal itself as fear of childbirth (FOC) in the peripartum period.^15^ Most women experience some degree of FOC, according to Wijma and Wijma.^16^ Severe FOC occurs when the delivery arouses fear to such a degree that it significantly impairs the woman's personal, social, relational, and/or professional life, and her willingness to become pregnant and/or her perceived competence to give birth. FOC is specifically related to labor and delivery, yet half of those with severe FOC also suffer from another kind of anxiety problem.^16^

A woman with severe FOC may be unable to objectively process information with respect to her upcoming childbirth, because in general, anxiety‐prone individuals are easily triggered by negative information,^17^ they may evaluate their situation in search of signs of danger and may attempt to avoid anything related to the fear‐inducing situation.^18^ For example, a woman may overestimate the risk of experiencing severe health problems either herself or by her child during birth or overestimate the risk of medical interventions in a hospital birth. Therefore, FOC will likely play a role in the preferred location for giving birth. Witteveen et al^19^ reported “more often pregnancy related anxiety in Dutch low risk women with planned hospital birth” compared to women with planned home birth, which concept roughly corresponds with FOC. However, in our study in 2005^7^ we did not find a difference in FOC between women preferring home or hospital birth. Moreover, we found increased FOC several weeks postpartum in the group of women who had, as a result of medical risk, undergone a compulsory move from home to hospital during labor. A woman who prefers home birth but ultimately gives birth in a hospital may have to deal with peripartum complications and giving birth in an environment that differs from her original preference, and this may exacerbate the level of FOC. However, the 2005 study was small, and meanwhile, in The Netherlands the home birth rate has rapidly declined. The purpose of this study is to test the following two hypotheses in a larger sample:

1. Women who prefer a home birth have a lower degree of FOC during pregnancy than women who prefer a hospital birth.
2. Women who give birth at a location other than their preferred location experience a higher level of FOC than women who give birth at their preferred location.

## 2 METHODS

**Figure 1:**
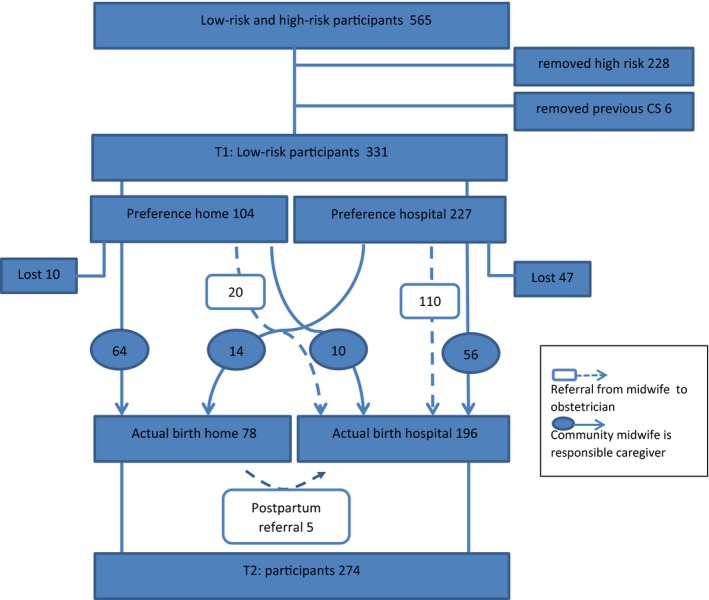
Flowchart showing the inclusion and exclusion of participants in the current study, The Netherlands, 2015‐2016

We included women who were in gestational week 30 of a singleton pregnancy and had a good command of the Dutch language. Using a Web‐based questionnaire, the total study sample consisted of 565 low‐risk and high‐risk participants, with a response rate of 68% of the 825 women who received a request by e‐mail. For the present study, we identified 331 women who were at low risk for pregnancy‐related complications and were under the care of a licensed midwife (see Figure 1). Women who had a high‐risk pregnancy or previously had delivered by means of cesarean were excluded.

### 2.1 Design

The prospective cohort study included 13 midwife practices in the southwestern part of The Netherlands, including both urban and rural areas. Participants were recruited from July 2014 through May 2015. At gestational week 20, eligible candidates received an information letter, including a link to the study's website. At gestational week 30 (defined here as T1), the participants received an e‐mail with a link to the first set of online questionnaires. Two months postpartum (T2), all participants who completed the questionnaire at T1 were e‐mailed with a link to the second set of questionnaires. As needed, up to five reminders were sent. Participants provided written informed consent for our researchers to analyze their obstetric files. The response rate at T2 was 83%. The study was approved by the Medical Ethics Committee of the Leiden University Medical Center (number P14.067).

### 2.2 Measures

The following sociodemographic data were collected at T1: age, marital status, education level, employment status, and country of birth. At T1 and/or T2, self‐reported obstetric data were collected: parity, referral (yes/no) and indication for referral, preferred and actual location for giving birth, transport during/after labor (yes/no and means of transportation if relevant), method of labor onset (natural/induced), use of pain relief (yes/no), delivery mode (vaginal birth/CS), and neonatal condition.

Preferred place of birth was determined using the question “If you could choose, would you prefer a home birth or hospital birth?” followed by an open question designed to determine the reasons for this preference. We developed a “congruence” variable by combining preferred and actual place of birth, with four possible outcome groups: home‐home, hospital‐hospital (preferred location congruent with actual birth location), home‐hospital, and hospital‐home (actual location incongruent with preferred location).

Referral was defined as handing over of the responsibility for the woman's obstetric care from the midwife to an obstetrician because of: (a) complications during pregnancy, labor, delivery, or within 2 hours postpartum; and (ii) the woman's request of pharmacological pain relief.

Fear of childbirth (FOC) was measured at T1 (version A) and T2 (version B) of the Wijma Delivery Expectancy/Experience Questionnaire (W‐DEQ), a 33‐item self‐assessment rating scale. The original Swedish version is well validated^20^, ^21^ and includes 33 statements with respect to giving birth with answers to be rated on a scale of 0‐5, yielding a final total score of 0 to 165. A higher W‐DEQ score indicates a higher level of FOC. A score ≥85 indicates severe FOC, whereas a score <85 represents a continuum ranging from no FOC to manageable FOC.^16^ Wijma et al previously reported that the internal reliability (Cronbach's alpha) of the W‐DEQ is 0.93 and 0.94 for versions A and B, respectively, and the split‐half reliability for both versions is >0.90.^20^ Consistent with this high degree of reliability, in our study Cronbach's alpha was 0.90 and 0.92 for versions A and B, respectively. Mentions of FOC in the following text refer to W‐DEQ scores.

FOC is a distinct psychological construct, separate from general anxiety.^20^ Therefore, at T1 we added the Hospital Anxiety and Depression Scale (HADS) to accentuate and statistically control for the difference between FOC, and general anxiety and depression.

HADS is designed to detect depression and anxiety among patients in a nonpsychiatric clinic^22^ and includes two 7‐item subscales (one for anxiety and one for depression), each with a total score ranging from 0 to 21. For each subscale, a score ≥11 is considered to represent clinically important signs of general anxiety or depression. In our study, Cronbach's alpha was 0.77 and 0.72 for the anxiety and depression subscales, respectively. A history of mental health problems (no/yes) was also asked for at T1.

### 2.3 Data analysis

For statistical analyses, we used IBM SPSS Statistics for Windows, version 23.0 (IBM Corp.). Groups were compared using the Pearson chi‐square test (for categorical variables) or the Student *t* test (for continuous variables). By scanning primary data for words and phrases most commonly used by respondents, the reasons for preference of home or hospital birth were evaluated.

In the following tests, the W‐DEQ score was used as a continuous variable. Differences in W‐DEQ scores between T1 and T2 were tested using a repeated‐measures ANOVA. Predictors for the preferred place of giving birth were evaluated using a logistic regression analysis.

A hierarchical multiple regression analysis was performed to analyze potential predictor variables of postpartum FOC. The mean W‐DEQ score (T2) was the dependent variable. We entered potential predictor variables of postpartum FOC in three consecutive stepwise blocks (Congruence groups, Obstetric characteristics, and Psychological precondition) to observe a weight shift in the previously entered general predictor variables on the addition of detailed personal predictors. This approach provides a more concise evaluation of the role of each variable from each domain. As referral to obstetrician‐led care is required in order to induce labor, to deliver using obstetric instruments, and/or to provide pharmaceutical pain relief, we only used the variable “Referral” in the analysis. The home‐home group was used as a reference group, as they had the lowest W‐DEQ scores.

## 3 RESULTS

At T1, 31% of the participants (104/331) reported that they preferred home birth, whereas the remaining 69% preferred a hospital birth (Figure 1). At T2, 28% of those still remaining in the study (78/274) had given birth at home, whereas the remaining 72% (196/274) had given birth at hospital.

Some commonly cited reasons for preferring a home birth were that respondents: wanted a familiar and comfortable setting, found it easier to relax at home, had heard positive experiences from family or friends, wished to avoid the presence of numerous medical staff while giving birth, and had a general fear of hospitals. Some commonly cited reasons for preferring a hospital birth were that respondents: felt safer with the availability of medical equipment, specialists, and pharmacological pain relief, felt their home was an inconvenient place to give birth, and wished to avoid the need for transport during labor in the event of complications.

A significantly higher percentage of women preferring home birth were multiparous, worked part time, and reported a history of mental problems than women preferring hospital birth (Table 1). In addition, the women preferring home birth had a lower degree of FOC, including a lower prevalence of severe FOC (*P* < .05) and lower mean W‐DEQ scores (Table 1).

**Table 1:** Characteristics of pregnant women at 30 wk gestation (T1) preferring home or hospital birth, The Netherlands, 2015‐2016

	Preference home n = 104 n (%) or mean ± SD	Preference hospital n = 227 n (%) or mean ± SD
Age		
≤25	7 (6.7)	21 (9.3)
26‐35	80 (77.0)	177 (78.0)
≥36	17 (16.3)	29 (12.7)
Educational level finished		
Elementary/high school	6 (6.0)	24 (10.6)
Vocational education (associates degree)	21 (20.0)	64 (28.2)
University (bachelor/master)	77 (74.0)	139 (61.2)
Country of origin		
The Netherlands	95 (91.3)	198 (87.2)
Other	9 (8.7)	29 (12.8)
Work**		
Full time	31 (29.8)	105 (46.3)
Part time	60 (57.7)	74 (32.6)
Unemployed/other	13 (12.5)	48 (21.1)
Marital status		
Married/cohabiting	102 (99.0)	224 (99.0)
Single mother	1 (1.0)	3 (1.0)
Mental problems now or in past*		
No	78 (75.7)	183 (86.7)
Yes	25 (24.3)	28 (13.3)
Parity***		
Nulliparous	41 (39.8)	149 (66.2)
Multiparous	62 (60.2)	76 (33.8)
Severe FOC*		
W‐DEQ <85	98 (94.0)	189 (86.0)
W‐DEQ ≥85	6 (6.0)	31 (14.0)
Mean W‐DEQ score**	55 ± 19.8	64 ± 18.3
HADS anxiety score	5 ± 3.4	5 ± 2.8
HADS depression score	3 ± 2.9	3 ± 2.6

Note

Numbers may not add to totals because of unknown responses.

* *P *< .05.

** *P *< .01.

*** *P *< .001.

Of the 274 women who completed the questionnaires at T2, a significantly higher percentage of women who preferred hospital birth were referred to an obstetrician (61%, 110/180) than women who preferred home birth (21%, 20/94) (*P* < .001). In addition, five of the women who gave birth at home were subsequently referred to hospital because of postpartum complications, three had preferred home birth, and the other two hospital birth. The main reason cited for referral during labor was to augment labor. In addition, 50% of all women being referred also received pain relief.

At T1, 11% of the entire cohort had severe FOC, whereas at T2, only 6% reported having severe FOC. The HADS anxiety and HADS depression scores correlated significantly with the W‐DEQ scores both at T1 (*r* = .31 and *r* = .28, respectively) and at T2 (*r* = .39 and *r* = .33, respectively).

Our results generally support our first hypothesis, as women preferring home birth had a significantly lower level of FOC at T1 than women preferring hospital birth (Table 1).

A logistic regression analysis revealed similar results. After adjusting for parity, education, and a history of mental health problems, women with higher FOC were more likely to prefer a hospital birth (OR: 1.02, n = 316, *P* < .001).

In contrast, our findings do not support hypothesis 2. We found that when prepartum FOC was taken into account, the presumed effect of “incongruence of preferred and actual place of birth” on postpartum measured FOC could not be demonstrated.

**Figure 2:**
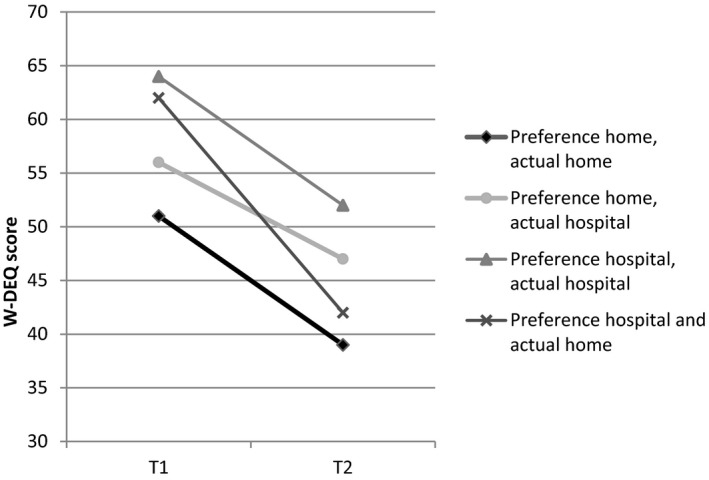
Mean W‐DEQ scores in the indicated groups based on their initial preference at T1 and their actual place of giving birth (reported at T2), The Netherlands, 2015‐2016

To reach this conclusion, first FOC (mean W‐DEQ scores) at T1 and T2 was examined for the four “congruence” groups (Figure 2). FOC in each group was higher at T1 than at T2 (*P* < .001). The only difference found between the four groups was that women in the home‐home group had a lower degree of FOC at both T1 and T2 than women in the hospital‐hospital group (*P* < .001) (repeated‐measures ANOVA using the Bonferroni post hoc correction).

The hierarchical multiple regression analysis (Table 2) also shows that the hospital→hospital group predicts higher postpartum FOC than the home→home group, whereas the two incongruent groups did not (block 1). However, after adding the obstetric variables to the model (block 2), the predictive role of preferred/actual place of birth is overtaken by obstetric predictors. The obstetric variables continue to have predictive value after adding psychological predictors (block 3). After including all variables, the model showed that the incongruence of preferred and actual place of birth did not predict postpartum FOC. However, a high degree of FOC at T1, high general anxiety at T1, a poor neonatal condition, and being referred to an obstetrician because of complications were related to higher postpartum FOC. This final model is statistically significant in predicting postpartum FOC (*P* < .001, adjusted *R* ^2^ = .27). This indicates that there are two major groups of women who are prone to postpartum FOC: those who have had medical complications concerning themselves or the baby, and those who already prepartum feared the delivery or had a more general anxiety.

**Table 2:** Correlation (partial *r*) of preferred‐actual place of birth with W‐DEQ scores two months postpartum, controlling for obstetric and psychological variables, The Netherlands, 2015‐2016

	Standard coefficient beta	*t*	Partial *r* Block 1	Partial *r* Block 1 + 2	Partial *r* Block 1 + 2 + 3
Block 1 preferred‐actual place of giving birth					
Home‐hospital	−.03	−0.48	.09	−.01	−.03
Hospital‐hospital	.02	0.31	.22***	.06	.02
Hospital‐home	−.03	−0.52	.03	.005	−.03
Block 2 obstetric variables					
Condition of the newborn (needed help/good)	−.18	−3.32		−.22***	−.20**
Referral from gestation week 30 till 2 h postpartum (no/yes)	.21	3.33		.21**	.20**
Parity (nulli/parous)	−.05	−0.81		−.14*	−.05
Block 3 psychological variables					
W‐DEQ T1	.30	5.00			.30***
HADS anxiety T1	.13	2.3			.14*
HADS depression T1	.003	0.05			.003
Adjusted *R* ^2^			.04	.17	.28

* *P *< .05.

** *P *< .01.

*** *P *< .001.

**Figure 3:**
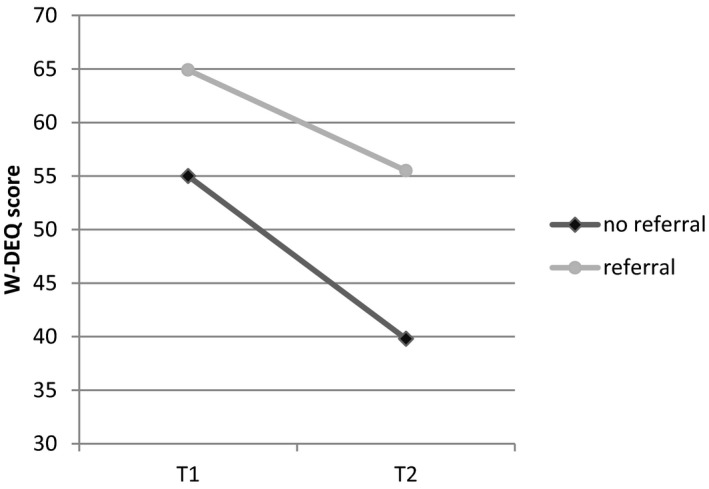
Mean W‐DEQ scores at T1 and T2, in women who remained under the care of their midwife (no referral) and in women who were referred to an obstetrician during pregnancy, during labor, or within 2 h of delivery, The Netherlands, 2015‐2016

In our final regression model, referral emerged as an important factor. Therefore, we examined the relationship between referral and both prepartum and postpartum FOC using a repeated‐measures ANOVA. We found that women who were referred to an obstetrician had a higher degree of FOC at T1 (*P* < .01), a smaller decrease in FOC measured at T2 (*P* < .05), and a higher degree of FOC measured at T2 (*P* < .01) than women not referred (Figure 3). Overall, we found that among the women who were referred, FOC measured at T2 was generally similar for the women who preferred home birth and the women who preferred a hospital birth.

Focusing on the 23 women who initially preferred home birth but were referred to an obstetrician, we found that ten were referred during pregnancy, ten were referred during labor (all transported with their own vehicle), and three were sent to hospital postpartum (of which two women were transported by ambulance). Among these 23 women, only two—both of whom were referred during pregnancy—had severe FOC measured at T2.

## 4 DISCUSSION

In support of our first hypothesis, our data show that women who prefer a hospital birth generally have a higher degree of FOC than women who prefer a home birth, irrespective of parity.

Consistent with previous reports,^23^, ^24^, ^25^, ^26^, ^27^, ^28^, ^29^ we found that women who prefer a hospital birth have a higher likelihood of being referred to an obstetrician and receiving medical interventions than women preferring home birth. As this group has an overrepresentation of women with a high degree of FOC, you may presume that these women desire a secure environment for giving birth, feel less confident in their ability to give birth,^30^ and generally have a strong desire for pharmacological pain relief,^31^ which all could be reasons for hospital birth preference. Importantly, the reasons for preferring a particular birth location and/or for referral to an obstetrician may be more complicated than meets the eye and could be motivated partly by psychological reasons.^32^

The preference for birth location in women with severe FOC may be based on anxiety‐inducing images instead of on rational considerations. This phenomenon of threat‐related attentional bias has been well documented in anxious individuals.^33^ Likewise, judgment of perceived risks is systematically biased in all women who have severe FOC, thereby causing an overestimation of the likelihood of critical—albeit rare—events such as losing the child, and/or an underestimation of the risks associated with less critical—but relatively common—adverse events such as the need to deliver by CS.^34^ Anecdotal stories from family, friends, and social media, and information available by means of the Internet can reinforce these biases. The woman's emotions connected to a desired or undesired outcome (ie, her intuitive evaluation) can lead to judgment bias with respect to the risks.^34^, ^35^ These emotions can be particularly strong and persistent in women who are prone to fear and can guide not only the decision with respect to where to give birth but also the interpretation of the childbirth experience.

Our results do not support our second hypothesis. Specifically, we found that giving birth at a location other than the woman's preferred location is not a predictor of a high level of FOC measured postpartum, after we adjusted for the variables “being referred” and “FOC during pregnancy.”

Our prediction model shown in Table 2 revealed that a high level of postpartum FOC is associated with high level of prepartum FOC (consistent with previous studies 27, 36), being referred to an obstetrician, and a poor neonatal condition. The latter two findings may be interpreted as signs of medical interventions and/or complications during labor, which have earlier been shown to increase the risk of postpartum FOC.^37^, ^38^

Although FOC, general anxiety, and depression were interrelated in our study, FOC clearly appears as a separate predictive construct. In our analyses, prepartum FOC holds up as a clearly discernable psychological variable in its own right, constituting a significant predictor of postpartum FOC even when general anxiety and depression are controlled for.

We found that women who initially preferred home birth but were referred to an obstetrician (in pregnancy or during labor) did not have a higher level of postpartum FOC than women being referred who initially preferred a hospital birth. This finding may seem surprising, as women who prefer home birth, but are referred to the hospital may need to adjust to the concept of receiving medical care in a more clinical setting. However, the women who initially preferred a home birth generally had lower FOC, which is strongly correlated with a lower level of FOC measured both while giving birth and postpartum.^36^ Women with a lower level of FOC generally feel more confident in their ability to give birth^30^ and may therefore be less focused on medical interventions such as pain relief. Rather, these women may focus more strongly on information that increases their confidence in giving birth, thereby helping them to reduce potential anxiety. Some of these women may also have been referred during pregnancy and have therefore had time to adjust to the change in location.

In addition to the above‐mentioned factors, the obstetric system itself also plays an important role. Most referrals to an obstetrician do not necessarily arise because of an acute situation^39^ and that was the case also in our study. Moreover, in the obstetric system where this study took place, the midwife responsible for the home birth accompanied the woman to hospital whenever possible. Other researchers have discussed how particularly fearful women can benefit from such continuous, familiar support.^40^, ^41^

### 4.1 Strengths and limitations

A strength of this study is its prospective design, which allowed us to follow women from gestational week 30 to 2 months postpartum. In addition, FOC was measured using the W‐DEQ, an established and broadly validated instrument used in many countries.

In our cohort, both prepartum FOC and postpartum FOC were generally low, as we intentionally selected women with a low‐risk pregnancy. Thus, the average level of FOC among our participants is not necessarily comparable to most studies with respect to FOC. Nevertheless, the high percentage of women who preferred home birth and indeed gave birth at home provides empirical insight into a cultural setting in which home birth is considered the standard option rather than an alternative. Conversely, this may be seen as a limitation with respect to the generalizability of our results, as this situation does not necessarily apply to countries that have a much lower rate of home birth rates than The Netherlands. An innate limitation of the study's design is that causality cannot be directly inferred from the identified associations.

### 4.2 Conclusions

We found that women who prefer home birth have a lower level of FOC than women who prefer a hospital birth. Interestingly, we also found that women who initially prefer a hospital birth are more likely to be referred to an obstetrician. Finally, we found that a high level of prepartum FOC, being referred to an obstetrician, and a poor neonatal condition predict a high level of FOC measured postpartum, whereas giving birth at a location other than the preferred location does not appear to affect postpartum FOC. Women with high FOC might give birth at home and in the hospital, but caregivers should pay extra attention to them because they are vulnerable to postpartum FOC, especially after a complicated birth requiring a referral.
