# Supplementary material for: Is fear of childbirth related to the woman’s preferred location for giving birth? A Dutch low‐risk cohort study
Source: Birth. 2019 Sep 24;47(1):144–52. doi: 10.1111/birt.12456 (PMC7065170; doi:10.1111/birt.12456)
Supplement: Supplementary file 1 [file BIRT-47-144-s001.docx]

Table 3, Supplemental information: Distribution of W-DEQ scores at T1 and T2 over the four congruence groups, subdivided according to parity and referral

| **Preferred/Actual location of giving birth** | **W-DEQ T1** | | **FOC T1**  **W-DEQ≥85** | **W-DEQ T2** | | **FOC T2**  **W-DEQ≥85** | **Parity** | **referral** | **W-DEQ T1** | | **W-DEQ T2** | |
| --- | --- | --- | --- | --- | --- | --- | --- | --- | --- | --- | --- | --- |
|  | n (274) | M  (SD) | n(%) | n  (270) | M  (SD) | n(%) |  |  | N  (271) | M min-max | N  (267) | M min-max |
| **Home/home** | 64 | 51.2  (16.6) | 1 (1.6) | 64 | 39.3  (18.8) | 1(1.6) | nulli | no | 22 | 58.1 20-89 | 22 | 42.8 12-84 |
|  |  |  |  |  |  |  |  | yes | 2* | 67.5 56-79 | 2 | 54.5 44-65 |
|  |  |  |  |  |  |  | parous | no | 35 | 46.7 12-75 | 35 | 35.6 8-85 |
|  |  |  |  |  |  |  |  | yes | 4** | 44.8 37-52 | 4 | 44.3 26-52 |
| **Home/hospital** | 30 | 56.9  (22.2) | 3 (10) | 29 | 46.6  (29.6) | 2 (6.9) | nulli | no | 3 | 71.3 64-81 | 3 | 49.7 41-65 |
|  |  |  |  |  |  |  |  | yes | 9 | 54.6 26-78 | 9 | 52.4 13-117 |
|  |  |  |  |  |  |  | parous | no | 7 | 58.1 24-114 | 7 | 33.7 9-60 |
|  |  |  |  |  |  |  |  | yes | 11 | 54.0 25-90 | 10 | 49.3 10-114 |
| **Hospital/hospital** | 166 | 64.3  (18.1) | 21 (12.7) | 164 | 52.1  (23.8) | 12 (7.3) | nulli | no | 29 | 66.9 46-99 | 29 | 45.3 14-117 |
|  |  |  |  |  |  |  |  | yes | 86 | 68.5 30-112 | 84 | 59.5 11-131 |
|  |  |  |  |  |  |  | parous | no | 26 | 48.7 24-101 | 26 | 36.5 10-83 |
|  |  |  |  |  |  |  |  | yes | 23 | 64.9 24-104 | 23 | 52.9 23-86 |
| **Hospital/home** | 14 | 58.9  (18.2) | 1 (7.1) | 13 | 40.7  (15.8) | 0 | nulli | no | 5 | 62.8 48-94 | 5 | 38.4 31-43 |
|  |  |  |  |  |  |  |  | yes | 1*** | 77 | 1 | 22 |
|  |  |  |  |  |  |  | parous | no | 7 | 50.1 27-72 | 6 | 47.5 25-82 |
|  |  |  |  |  |  |  |  | yes | 1**** | 83 | 1 | 30 |

*1.complication resolved, back to midwife before giving birth 2.postpartum hemorrhage **1.manual placenta removal , 2.3th degree rupture, 3. and 4.complication resolved, back to midwife before giving birth

*** postpartum hemorrhage ****3th degree rupture
